# Preclinical studies of sex differences: a clinical perspective

**DOI:** 10.1186/s13293-016-0061-2

**Published:** 2016-01-22

**Authors:** Rhonda Voskuhl

**Affiliations:** Jack H. Skirball Chair in MS Research, Multiple Sclerosis Program, Department of Neurology, UCLA School of Medicine, Neuroscience Research Building 1, Room 475, 635 Charles E. Young Drive South, Los Angeles, CA 90024 USA

**Keywords:** Sex differences, Translational research, National Institutes of Health

## Abstract

The new policy from the National Institutes of Health to encourage grant applicants to consider studying both females and males in preclinical biological experiments has been met with support and opposition. Here, we will discuss implications of preclinical studies of sex differences on clinical research.

## Letters to the Editor

It is now over a year since the announcement of a new National Institutes of Health (NIH) policy to encourage applicants to consider studying both females and males in preclinical biological experiments. The policy would prevent researchers from assuming no sex difference or ignoring one sex entirely [1]. Implementation of this policy is now beginning. It has been supported by some [2], while others oppose [3]. Should all researchers be expected to assess for sex differences [4]? Here, I offer a clinical perspective.

Sex differences in health and disease are numerous. There are sex differences in neurodegenerative, cardiovascular, and autoimmune diseases, to name a few [5]. Clinical observations of sex differences in disease should be viewed as precious clues in the search for new treatments to protect from disease, since they are naturally occurring disease modifiers [6]. Preclinical research on animals is essential for disentangling various mechanisms underlying sex differences in human diseases, revealing candidate targets for ultimate translation back to humans in a “bedside to bench to bedside” approach. We have recently employed this strategy to investigate the protective effects of an estrogen treatment in women with multiple sclerosis [7].

Arguments against basic biological research on sex differences in animals have included the idea that cells studied in the culture dish do not function like cells in the human body and that mice differ from humans [3]. However, those limitations are true for all preclinical research, not just sex differences. No preclinical model perfectly simulates all aspects of human disease. Each model is chosen based on a specific question, and several different models are used to provide comprehensive insight. Why should the study of the biology of sex differences be held to a different standard?

Another rationale for not doing biological sex differences research has been that there are gender-related social factors in humans that are poorly modeled in preclinical research [3]. Most clinical observations in humans are the result of both biological and social factors, nature and nurture. There are social and cultural differences affiliated with age and race. Does this preclude the study of biological effects of aging or race? No. Sex, age, and race are each important variables that impact disease, and understanding both the biological and social factors is needed for optimal treatments in each population.

Let us now look beyond the first step the NIH has proposed that encourages researchers to consider sex as a biological variable in their systems. In the years ahead, this will result in many new observations of sex differences. When you look, you find. But to show that sex differences exist is the beginning, not the end. A second step is needed. Support for research to understand the mechanistic basis for observed sex differences will be required to capitalize on initial observations and translate them back to human disease. If step one is taken without step two, a bottleneck of observations regarding sex differences will accumulate. Both steps are needed for ultimate translation back to the clinic.

In summary, an inconvenient truth for some seems to be that biological sex differences exist in many systems. Speaking as a feminist, sex differences do not imply inequality of the sexes. Speaking as a scientist, knowledge is always the path forward. Speaking as a physician, how can we justify ignoring a major disease modifier to patients? Rather, we must embrace the study of sex differences without political bias or fear of misinterpretation in the pursuit of health for man- and womankind.

## Abbreviations

NIH: National Institutes of Health
